# Perceived competence in ice hockey and its associations with relative age, early sport specialization, and players’ position

**DOI:** 10.3389/fpsyg.2024.1336529

**Published:** 2024-01-25

**Authors:** Vincent Huard Pelletier, Jean Lemoyne

**Affiliations:** ^1^Laboratoire de Recherche sur le Hockey de l’UQTR, Université du Québec à Trois-Rivières, Trois-Rivières, QC, Canada; ^2^Département des sciences de l’activité physique, Université du Québec à Trois-Rivières, Trois-Rivières, QC, Canada

**Keywords:** sport development, self-enhancement, perceived competence, organized sports, adolescence, relative age effect, sport expertise

## Abstract

**Introduction:**

Ice hockey is a sport that has gained much attention in recent times, particularly concerning the development of young players. In the domain of youth sport development, one significant factor that must be considered is the perceived competence of players. This variable is closely linked to positive psychological outcomes and sustained practice. However, there is a lack of understanding about how other important developmental factors such as age, early sport specialization, players’ position and relative age affect players’ perceived competence. Therefore, the objective of this study is to explore the relationships between these developmental factors, perceived ice hockey competence and a global measure of perceived sport competence.

**Methods:**

Data was drawn from 971 players (14.78 ± 1.61 mean age), who completed on-line questionnaires, from which we conducted path analyses involving all variables.

**Results:**

Younger players tend to display higher perceived competence scores than older players. Additionally, players who opted to specialize earlier also reported higher perceived competence. Furthermore, forwards and defensemen had differing perceptions of their competence, which was in line with their respective roles on the ice. The study also showed relative age effects, in which players who were born earlier relative to the selection period tend to perceive themselves more advantageously in three components of perceived competence.

**Discussion:**

Based on these findings, several recommendations are proposed for coaches and decision-makers to encourage the positive development of ice hockey players. The study highlights that ice hockey-specific competencies are influenced by various factors, such as early sport specialization, relative age effect, player age, and position.

## 1 Introduction

Organized sport occupies a prominent place in society and is adolescents’ practice of choice in terms of physical activity (Malm et al., 2019). In fact, nearly 60% of Canadian youth aged between 5 and 17 participate in at least one organized sport, and 74% were involved in it before the COVID-19 pandemic (Kuzik et al., 2023). One of Canada’s most important organized sports is ice hockey, with over 500,000 registrations in minor ice hockey and numerous successes in international competitions (Hockey Canada, 2023). Because of its growing popularity and young players’ eagerness to achieve high-performance levels, youth sports are rapidly becoming professionalized (Camiré and Santos, 2019), which can lead to external pressures from social agents such as parents and coaches (Todd and Edwards, 2021). However, studies on soccer reveal that attendance at a sports academy at a young age does not seem to have an impact on professional success, nor does performance before the age of 14 (Leyhr et al., 2018; Dugdale et al., 2021). Recently, governing bodies have focused on creating environments more conducive to the players’ long-term development regarding technique, tactics, self-confidence, and sustained sports participation. However, coaches have identified many challenges regarding the complexity, applicability, and compatibility of these measures with the realities of the playing field (Beaudoin et al., 2015). Obstacles to young athletes’ continued involvement in sports include time conflicts with other activities, lack of motivation, negative relationships with coaches and low perceived competence (Balish et al., 2014). The latter has been recognized for several decades as an important psychological construct in the development of young people, as it enables long-term practice and well-being (Harter, 1978).

As defined by Shavelson et al. (1976), perceived competence refers to an individual’s perception of their abilities in a particular performance domain. This concept is central to various theoretical models used in sports and physical activity research, including the Self-Determination Theory (Deci and Ryan, 2008) and the Youth Physical Activity Promotion Model (Welk, 1999). A positive perception of perceived sports competence is associated with high levels of intrinsic motivation (Rodrigues et al., 2021), long-term commitment to sports (Feltz and Petlichkoff, 1983) and achievement of the highest performance standards (Feltz, 1988). Another framework that underlines the importance of perceived competence is the Personal Assets Framework (Côté et al., 2014). Tenants of this model stipulate that perceived competence is fostered by the quality of the environment in which young athletes evolve.

Throughout their formative years, individuals’ self-assessment of their ability as players undergoes a transformation. In early childhood, it can be challenging for young athletes to distinguish between competence, effort, achievement, luck, ability and task difficulty when assessing oneself. Nevertheless, as they enter adolescence, their ability to evaluate their own competencies progresses significantly (Horn et al., 1993; Horn, 2004). Adolescents start using a broader range of resources to evaluate themselves (e.g., feedback from peers and teammates) and can differentiate more effectively between these resources. Moreover, they become more proficient at using self-determined performance standards, such as achieving self-set goals, and rely more on internal and psychologically based information like feelings of self-confidence or personal efficacy. While adolescents still compare themselves to their peers within the competitive sport setting, they are now capable of doing so in relation to a more extensive, extended peer group they may not personally know (e.g., “How do I rank within the state or nation in my age group?”). The current literature indicates that young athletes’ sense of competence tends to become more accurate during adolescence and diminishes as well (Tubić and Đorđić, 2015). However, no studies to date have examined perceptions of competence during adolescence in ice hockey, a sport with a significant drop-out rate in adolescence (Lemez et al., 2014).

Despite the relevance of perceived sports competence in sports development models, competence is generally measured only with items that focus on sports in general. This makes sense, of course, when measuring a general population in which a multitude of sports can be practiced (Harter, 1978; Fox and Corbin, 1989). Rottensteiner et al. (2015) however, argues that an athlete’s perceived competence in a particular discipline is associated with increased participation in that discipline, underlining the importance of a sport-specific measure under study. Lemoyne et al., 2015 also offered a good example of such associations with a cohort of college students. As a result, efforts have been made in recent years to measure perceptual competence for a specific sport, such as swimming (Marsh and Perry, 2005), gymnastics (Kipp and Weiss, 2013), or soccer (Forsman et al., 2016). More recently, the Self-Perceived Ice Hockey Competence Scale was developed and validated with adolescent hockey players (Huard Pelletier and Lemoyne, 2023). This study showed that ice hockey perceived competence is characterized by six dimensions: skating, strength and power, tactics, offensive abilities, coachability and resilience. Skating is the ability to move with ease on the ice rink and is increasingly important in modern ice hockey. The “strength and power” dimension refers to the ability to produce strength, whether in shots, in one-on-one battles or by other types of physical involvement on the ice (e.g., body checks). Tactics involve making the right decisions with and without the puck and applying the coach’s play system. Offensive abilities include goal-scoring skills such as puck handling and shooting accuracy. Coachability refers to leadership skills and gestures that promote positive interactions with teammates and coaches, while resilience reflects the ability to cope with adversity and difficult situations.

In sports, there is great interest in having young athletes compete at the highest level before reaching adulthood, whether at the amateur or professional levels (Malina, 2010). This seemingly innocuous phenomenon is not without consequences for youths and their behavior. Recent years have witnessed a more significant investment of free family time in organized sporting activities and the professionalization of sports (Feeley et al., 2016) to improve children’s performance and enable them to out-perform others (Sturm, 2005). This may have been exacerbated by success stories like those of Andre Agassi and Tiger Woods, who were involved intensively in sport specialization behaviors at a very young age and quickly entered the world’s elite (Farrey, 2008; Agassi, 2011). Indeed, this trend toward early sport specialization has become increasingly pronounced in recent decades (Smith, 2015). The construct of sport specialization was originally developed by Wiersma (2000) and is defined as participation in a single sport at a high intensity of training and competition. Early sport specialization (ESS) definition was refined more than a decade later by Jayanthi et al. (2013) who proposes ESS as an “intense training in a single sport to the exclusion of all other sports.” Laprade et al. (2016) went a step further, defining ESS as “the decision to focus on a specific sport for at least 8 months a year before the age of 12 and to participate in specific training and competitions throughout the year, often to the detriment of exploring other sports.” Recently, Kliethermes et al. (2021) underscored the lack of consistency in the definition and measurement of the ESS concept. In view of the multiple determinants and complexity of ESS, each of its characteristics’ merits attention. Furthermore, this approach is often motivated by the desire to progress rapidly and maximize the chances of success in a given discipline (Normand et al., 2017); the result is increasing reports of negative effects such as a greater number of overuse injuries (Bell et al., 2018) and psychological issues leading players to quit the sport (Keegan et al., 2010). In Canadian youth hockey, early sport specialization is prevalent across all age groups and playing levels, regardless of gender (Huard Pelletier and Lemoyne, 2022). It’s important to determine whether specialization impacts players’ perceived competence. However, different studies have produced conflicting results. One study suggests a connection between specialization and perceived physical self-concept (Huard Pelletier and Lemoyne, 2020), while another shows opposite results for perceived competence, specifically in ice hockey (Huard Pelletier and Lemoyne, 2022). According to a review by Mosher et al. (2022), we must now attempt to better understand the mechanisms behind the potentially harmful effects of early sport specialization. For example, do the negative psychological effects of ESS stem from the demands of early rigorous physical conditioning, participation in specialized off-season camps, or the fact that playing ice hockey precludes participation in other sports? Furthermore, the development of the perceived Ice Hockey Competence scale has now made it possible to measure the links between ESS and ice hockey competence, which was not formally done in the past. Could specialized players feel competent in their chosen sport, where they invest most of their time, but feel much less so in sports overall?

Another construct that may influence ice hockey players’ perception of competence but has rarely been measured in the scientific literature to date is the position the player evolves in. Indeed, forwards and defensemen have significantly different tasks on the ice; the primary mandate of forwards is to score goals, while that of defensemen (and goaltenders) is to defend, or prevent the opponents from the same outcome. In the past, defenders were shown to be generally taller, heavier and more powerful in terms of upper-body muscles, while forwards were smaller, faster and more agile (Geithner et al., 2006; Kutáč and Sigmund, 2015). However, a recent study of non-professional ice hockey players aged 17 to 23 reported no significant anthropometric differences between forwards and defenders (Czont et al., 2023). Technically, there are important differences in the way forwards and defenders move around the rink; forwards make more quick turns, accelerate and move more often at high speed, while defenders spend more of their time skating backwards and must often change direction (Montgomery et al., 2004). Furthermore, the birth distribution seems to differ according to the players’ position, as demonstrated in ice hockey (Grondin and Trudeau, 1991) and junior soccer (Romann and Fuchslocher, 2013). Additionally, there are trends toward early position-specific specialization in sports such as baseball (Ogden and Warneke, 2010), volleyball and basketball (Horn, 2015), all technically demanding sports where the athlete’s size is essential. The aforementioned paper may also provide insights into birth distribution and players’ positions. In this regard, it seems probable that forwards and defensemen might be different in terms of their tasks and physical condition. This begs the question of whether a player’s position impacts their self-perception as an athlete and, if so, how.

When it comes to organized sports, access to the highest competitive levels is affected by the relative age effect (RAE). According to Cobley et al. (2009), RAE refers to the observation that, for a particular age category, children born earlier in the selection period tend to be over-represented in elite sports teams compared to those born later in the year (Cobley et al., 2009). The reason is that categories are divided based on birth dates in organized sports and schools. The biased selection of athletes is well-documented and can affect players’ development opportunities. Musch and Grondin (2001) proposed four mechanisms contributing to Relative Age Effects (RAEs) in organized sports. They include (1) depth of competition, where RAEs are more significant when many athletes compete for fewer roster positions; (2) physical development differences, where RAEs are more prominent in sports that prioritize players who experience earlier physical growth; (3) cognitive differences, where RAEs result from psychological advantages among relatively older athletes; and (4) experience, where relatively older children have more sports practice than younger ones. Those advantaged by the relative age effect have access to better coaches and higher levels of competition (Baker et al., 2010). This phenomenon can be described as the *Mathew Effect*, where the rich get richer, and the poor stay poorer (Hancock et al., 2013). Secondly, relatively older players have increased expectations from coaches, which helps them develop and is known as the *Pygmalion effect*. Finally, athletes with a relative age advantage can develop better self-perceptions (Lemoyne et al., 2021), leading to better performances. This phenomenon is known as the *Galatea effect*. It’s unclear, however, whether these young athletes also perceive themselves as better in specific aspects of ice hockey competence.

Exploring the connections between perceived competence, early sport specialization, position played, and relative age effect in youth hockey is paramount. However, the current literature lacks a conclusive understanding of these links, largely due to the absence of validated questionnaires on perceived ice hockey competence. Additionally, the specific aspects of early sport specialization that impact perceived competence among young ice hockey players remain unclear. For instance, it is unknown whether playing ice hockey for more than 8 months annually at a young age or participating in off-season development camps significantly affects a player’s perceived competence. Furthermore, a lack of empirical data allows us to affirm if forwards feel more competent than defensemen, and vice versa. By properly understanding the intricate relationships between these variables, we can evaluate the potential effects of early specialization and age-based selection on the development of young ice hockey players. More specifically, this study aims to model the impact of age categories, early sport specialization, player position and birth quartiles on ice hockey-specific perceived competence and perceived sport competence. Based on the existing literature, four hypotheses can be put forward:

1. Younger players should display higher levels of ice hockey competence;
2. Specialized players should have more negative self-perceptions. Inversely, less specialized players should have similar or even stronger self-evaluation of their global perceived sport competence;
3. There should be differences in perceived competence according to players’ position;
4. There should be relative age effect on players’ perceived competence.

## 2 Materials and methods

### 2.1 Sample and data collection

Participants were recruited with the collaboration of the Quebec Ice Hockey Federation and approved by the board of ethics of the researchers’ institution (CER-21-27507.04). Invitations were sent directly to ice hockey program directors across the province. The directors of 29 organizations accepted; they were subsequently contacted for an information session and invited to complete an online questionnaire (using the Qualtrics platform). In the participating organizations, each program director sent invitations to the players of 54 teams across the 29 organizations. To be eligible, participants had to be between 12 and 17 years old and registered with the provincial ice hockey federation. This age bracket made it possible to include most players at the U13, U15 and U18 levels, corresponding to the age of students in Quebec high schools. We informed the players of the project and obtained their written consent or that of their parents (for players under 14). It should be noted that data collection was carried out exclusively in French, the official language of the population under study.

### 2.2 Measurement

#### 2.2.1 Perceived competence

We measured perceived ice hockey competence with the Self-Perceived Ice Hockey Competence Scale (Huard Pelletier and Lemoyne, 2023). This questionnaire was previously validated in French and using 5-point Likert scales (from 1 = does not represent me at all, to 5 = represents me perfectly). The questionnaire comprises 6 subscales: skating (4 items; I am a fast skater), strength and power (4 items; I am physically strong), tactics (4 items; I can apply the game systems without problem), offensive abilities (4 items; I am efficient in the offensive zone), coachability (3 items; I have a good work ethic that helps me to perform) and resilience (3 items; I remain confident even if my playing time decreases). This questionnaire provides a good conceptualization of the perceived competence specific to ice hockey. It also displays good reliability (McDonald’s Omega ω varying from 0.74 to 0.84) and good temporal stability (intraclass correlation coefficient all >0.87). The items of this instrument can be seen in Supplementary File 1. We also measured perceived sports competence (PSC) using three 5-point Likert-type items taken from the French short version of Corbin’s Physical Self-Perception Profile (Maïano et al., 2008) (e.g., *Inventaire du Soi Physique*) with the following wording: (a) *I find that all sports are easy for me*, (b) *I find that I’m good in all sports*, and (c) *I do well in sports*. The subscale showed good internal reliability (ω = 0.94).

#### 2.2.2 Player’s age and position

Players’ age was categorized in three different age-groups in which they play: (e.g., U13: 12–13 years = 1, U15: 14–15 years =2 and U18: 16, 17, 18 years = 3). Players’ position was dichotomized: forwards (1) and defensemen (2). Both variables were then treated as categorical variables.

#### 2.2.3 Early sport specialization

Early sport specialization indicators were measured retrospectively with three questions inspired by LaPrade et al. (2016) definition and previous ice hockey studies (Huard Pelletier and Lemoyne, 2020 2022) using a Likert scale with three response options. It’s worth noting that since the ice hockey season spans at least 8 months, athletes were not required to specify this in the questionnaire. The first question was “I feel that playing ice hockey prevented me from playing another sport” (1 = Definitely, 2 = Very little, 3 = Not at all). The second and third questions were “At what age did you start summer strength and conditioning specifically for ice hockey on a regular basis” and “At what age did you start participating in summer development hockey-specific activities on a regular basis” (1 = From 4 to 8 years old, 2 = From 9 to 11 years old, 3 = From 12 to 16 years old, 4 = Never/not yet). Internal consistency for the ESS measures suggests acceptable reliability (ω = 0.71). We then calculated a composite score (mean) by regrouping each ESS indicators for the following analyses.

#### 2.2.4 Relative age

The athlete’s month and year of birth were requested in the questionnaire. Months were then categorized according to birth quartile (1 = January–March, 2 = April–June, 3 = July–September, 4 = October–December), which was treated as a categorical variable. Birth quartiles were congruent with the Canadian Ice Hockey Federation guidelines for defining age group categories. To prevent the potential bias distribution of birth quartiles (e.g., make sure that the actual sample is similar to population), we followed Delorme and Champely (2015) recommendations by analyzing 2024’s province of Quebec birth rates, because it corresponds to the median age of our sample (Statistique Canada, 2018). We found no significant differences in birth quartile distributions.

### 2.3 Statistical analysis

The following analyses were carried out with Mplus (version 8). Descriptive statistics and data distribution (kurtosis, skewness) were measured to determine whether the sample’s assumption of normality was violated. After analysis, it was concluded that the sample was not normally distributed, so the maximum likelihood Robust (MLR) modeling procedure was used to evaluate the path analysis (Yuan and Bentler, 2007). To address the four research objectives, a path analysis was conducted to model the possible influences of ESS, age category, player position and birth quartile on the six dimensions of perceived ice hockey competence and perceived sport competence. Since there was not a specific model to test involving the constructs under study, the path analysis approach allows more flexibility for testing associations between all independent variables in a same model, and their links with each component of perceived competence (global or specific to ice hockey). Each construct, except for ESS, was treated as an observed variable in the model. Consequently, this means that path coefficients that involve ESS construct with component of perceived competence were treated as continuous (e.g., higher coefficient means strong associations). For the categorical variables (e.g., Age, RAE, players’ position), paths coefficients were determined form each factor’s corresponding reference category. Model interpretation was based on the most common fit indices. The chi-square statistic (χ^2^) indicates the level of model fit. To estimate the quality of the indices, we used the Comparative Fit Index (CFI) and the Tucker-Lewis Index (TLI). Estimation errors were estimated using the Rooted Mean Square Error of Approximation (RMSEA) and the Standardized rooted mean square residual (SRMR). To interpret the indices, we followed the suggestions of Hu and Bentler (1999): 1- a non-significant value of χ2 (goodness-of-fit); CFI and TLI > 0.950; RMSEA and SRMR <0.07. The LaGrange Multiplier (LM) test was also considered, but only for modifications supported by theoretical assumptions. To verify if the new model was significantly better than the previous one, Satorra and Bentler (2001) scaled chi-square difference (SBΔ*χ*^2^) test was used.

## 3 Results

### 3.1 Sample

Table 1 describes the study’s sample with regard to all variables. Of the 1,058 players contacted, 971 (92%) agreed to answer the questionnaire. Once goaltenders were excluded, the sample size decreased to 864 (76%). All participants were males between 12 and 18 years old, with a mean age of 14.78 ± 1.61; they were selected from all development networks in the area of the study. The sample comprised 24.9% U13 players, 41.6% U15 players, and 33.6% U18 players. Most in the sample (59%) played as forwards, while 36% played as defensemen. This was expected because all teams align more forwards than defensemen. Only 5% of participants (n = 43) neglected to mention their playing position. Regarding birth quartiles, 33.3% (n = 285) were born in the 1^st^, 24.3% in the 2^nd^ (n = 207), 20% in the 3^rd^ (n = 172), and 20.4% in the 4^th^(n = 173). In this regard, a significant relative age effect was observed with the current sample (χ^2^_(df)_ = 40.76_(3)_; *p* < 0.001).

**Table 1:** Means, standard deviation, and inter-items correlation.

Variables (*n* = 774)	M ± SD	1	2	3	4	5	6	7
1. Skating	4.00 ± 0.75	1						
2. Strength	3.93 ± 0.72	0.436**	1					
3. Tactical	4.18 ± 0.60	0.540**	0.517**	1				
4. Offensive	3.87 ± 0.65	0.491**	0.408**	0.469**	1			
5. Coach	4.15 ± 0.68	0.500**	0.438**	0.623**	0.430**	1		
6. Resilience	4.06. ±0.68	0.436**	0.386**	0.518**	0.331**	0.521*	1	
7. PSC	2.08 ± 0.76	0.627**	0.491**	0.586**	0.528**	0.558**	0.418**	1

### 3.2 Model estimation

Despite acceptable fit indices (*χ*^2^_(df)_ = 47.72_(23)_, *p* = 0.002: CFI = 0.990, TLI = 0.966; RMSEA = 0.037), the results of the LM test suggest the addition of two covariances (age categories with Birth Quartile; age categories with engagement in hockey specific activities). Both additions produced a final model that displayed a better fit (*χ*^2^_(df)_ = 26.24_(22)_, *p* = 0.241: CFI = 0.998, TLI = 0.994; RMSEA = 0.015). In fact, these additions resulted in significant improvement of the proposed model (ΔSB*χ*^2^ = 21.48_(1)_, *p* < 0.001). All model standardized estimates are presented in Table 2 at the end of the Results section.

**Table 2:** Age, ESS, player’s position, birth quartile, and perceived competence.

Exogenous variable →	Endogenous variable	Estimate	Standard error	*p*-value
Age category^1^	Perceived skating	−0.090*	0.037	0.016
ESS		0.144**	0.042	0.001
Position^2^		0.001	0.055	0.991
Birth quartile^3^		0.018	0.024	0.436
Age category	Perceived strength and power	0.003	0.035	0.924
ESS		0.272**	0.037	0.000
Position		0.111*	0.051	0.030
Birth quartile		−0.063**	0.023	0.007
Age category	Perceived tactical abilities	0.049	0.032	0.121
ESS		0.192**	0.035	0.000
Position		0.138**	0.045	0.002
Birth quartile		−0.047*	0.020	0.018
Age category	Perceived offensive abilities	−0.057	0.034	0.092
ESS		0.209**	0.035	0.000
Position		−0.328**	0.046	0.000
Birth quartile		−0.017	0.020	0.404
Age category	Perceived Coachability	0.011	0.034	0.743
ESS		0.226**	0.042	0.000
Position		0.005	0.053	0.925
Birth quartile		−0.048*	0.023	0.037
Age category	Perceived Resilience	−0.079*	0.034	0.021
ESS		0.086*	0.036	0.018
Position		0.019	0.053	0.721
Birth quartile		−0.016	0.022	0.483
Age category	Global PSC	−0.017	0.035	0.624
ESS		0.226**	0.040	0.000
Position		0.002	0.050	0.967
Birth quartile		−0.029	0.022	0.186

*p < 0.05; **p < 0.01. ESS: Early sport specialization.

1 Age; U13 = 1, U15 = 2, U18 = 3. Negative coefficients mean lower scores for older group.

2 Position; Forwards = 1, Defensemen 2. Negative coefficients mean stronger scores for forwards.

3 Birth Quartiles; 1 = Q1, 2 = Q2, 3 = Q3, 4 = Q4. Negative coefficients mean lower scores for Q4.

#### 3.2.1 Objective 1 – age and perceived competence

As Table 2 demonstrates, older players (U18 = 3) displayed lower scores in two components of ice hockey perceived competence: perceived skating abilities (β = −0.090, *p* = 0.016) and resilience (β = −0.079, *p* = 0.021). The negative loading suggests that older age groups tend to display lower scores for these two sub-dimensions. Interestingly, this tendency was also observed with regard to perceived offensive abilities (β = −0.057, *p* = 0.092). No other age-related differences were noted.

#### 3.2.2 Objective 2 – ESS and perceived competence

Table 2 shows that ESS tends to have significant, favorable associations with all components of perceived competence. Significant regression coefficients were observed for perceived skating abilities (β = 0.144, *p* = 0.001), perceived strength and power (β = 0.272, *p* < 0.001), perceived tactical abilities (β = 0.192, *p* < 0.001), perceived offensive abilities (β = 0.209, *p* < 0.001), coachability (β = 0.226, *p* < 0.001), resilience (β = 0.086, *p* = 0.018) and PSC (β = 0.226, *p* < 0.001). In summary, results suggest that players who tend to be more specialized in their sport (e.g., high ESS scores) tend to have stronger perceptions of their competencies.

#### 3.2.3 Objective 3 – player position and perceived competence

Results in terms of players’ position reveal some differences. In general, they suggest that player position is significantly associated with three components of perceived competence in ice hockey. Some loadings, however, showed specific position-related differences. Defensemen (higher category) reported higher scores in perceived strength and power (β = 0.111, *p* = 0.030) and perceived tactical skill abilities (β = 0.138, *p* = 0.002), while forwards, conversely, tend to display higher scores for offensive skills (β = −0.328, *p* = 0.037).

#### 3.2.4 Objective 4 – relative age effect on perceived competence

Table 2 suggests the presence of a relative age effect on three of the six ice hockey competencies. In fact, being born in a later quartile (e.g., Q4 versus Q1) suggests significant, negative associations for three dimensions of perceived competence: strength and power (β = −0.063, *p* = 0.007), tactical skills (β = −0.047, *p* = 0.018) and coachability (β = −0.048, *p* = 0.037). There was no significant difference for the other perceived competence dimensions.

## 4 Discussion

This study’s objective was to analyze the associations between relative age effect, early specialization in sports, player position choice and perceived competence in Canadian men’s minor ice hockey. To our knowledge, this is the first study to link aspects of sports development (age, ESS, player position, REA) with perceived sport-specific competencies in young people. Following a thorough review of the relevant literature, four objectives and hypotheses were formulated and examined. The first objective was to measure perceived competence as a function of players’ age. According to our results, players who evolve in older age group categories tend to have lower perceptions of their offensive and skating abilities and their perceived resilience, confirming hypothesis 1. Such a tendency was expected because, in late adolescence, players have access to more resources for self-comparison and can better identify their weaknesses (Horn, 2004). By the same token, players in older categories may have a more accurate or informed assessment of their skills. Similar tendencies were observed for Perceived strength-power and PSC, although not statistically significant. Tactical skills and coachability, on the other hand, tend to increase slightly, but not significantly, in the current sample. The reason may be that players deepen their tactical understanding of the game during adolescence and feel more at ease after a few years of learning. In addition, they begin to understand the importance of a work ethic and what their coaches expect of them.

The second objective was to measure the potential influence of early specialization on perceived competence, either in a specific context (e.g., ice hockey) or from a general perspective (perceived sports competence). Our results show that early sport specialization appears to be positively related to both perceived ice hockey competence and global perceived sport competence, refuting hypothesis 2. Although such results may come as a surprise given the potentially negative impact of ESS on perceived competence (Gould, 2010), it’s reasonable to believe that time invested in a preferred sport may have a positive impact on self-perceptions of one’s athletic skills, suggesting a specific relationship between perceived competence and the physical activity corresponding to the targeted competence. This relationship, moreover, is supported by Lemoyne et al. (2015) using samples of college students.

One of the reasons why ESS is associated with a more positive perception of skating abilities as well as strength and power might be athletes’ investment of time and effort during the off-season. Indeed, improvements and efforts in strength and conditioning during the off-season are associated with more positive perceptions of the physical self in university athletes (Jones et al., 2010). Additionally, improvements in ice hockey players’ physical fitness have a direct impact on skating performance (Delisle-Houde et al., 2019) and ice time (Delisle-Houde et al., 2018). Professionally supervised strength and conditioning training for young people is beneficial and recommended for enhancing motor skill development, reducing injury risks and improving self-esteem; thus, the results of this study are consistent with this approach (Faigenbaum et al., 2009). The adoption of a sport practice similar to ESS also seems to be associated with more positive perceptions of tactical and offensive skills. We can presume that young athletes who take part in several ice hockey-specific development camps often rub shoulders with quality coaches who help them better understand the tactical aspect of the sport, even though no studies have examined the content of these camps to date. However, it’s possible that athletes who choose to spend more time with qualified coaches during the summer to improve their shooting, passing and puck-handling skills likely feel more competent regarding the offensive dimension as well (Aalto and Räihä, 2012). This study also highlights positive associations between ESS and the two psychosocial dimensions of ice hockey competence: coachability and resilience. The reason may be that young people who opt for specialization behaviors undergo high volumes of on-ice and strength-conditioning (Bell et al., 2018) and must therefore develop a higher-than-average work ethic (coachability) and perseverance (resilience) to sustain this practice over time. It is generally accepted that ESS brings players into contact with many high-level athletes, which may be conducive to more negative self-perceptions (Gould, 2010). However, based on the results of this study, we believe that exposure to role models who perform and invest a great deal of time in their chosen sport may also benefit young ice hockey players by enabling them to learn various life skills (communication, cooperation, goal setting, leadership) that increase their feeling of competence (de Subijana et al., 2022). By definition, specialized young people opt for the intensive practice of one sport to the detriment of others (Bell et al., 2021). Therefore, they may be expected to feel competent in their chosen sport, but much less so regarding other sports in general. However, the results of this study do not support this view. This may be partly because ice hockey is a difficult sport requiring many different skills (speed, power, agility, decision-making) that can be transferred to other sports (Vigh-Larsen and Mohr, 2022).

The third objective, to measure potential differences in perceived competence based on players’ positions, gave rise to some interesting conclusions and confirmed the third hypothesis. Results indicate that defensemen perceived themselves to be more powerful and more competent in playing team systems (e.g., tactical skills). Forwards, on the other hand, showed higher perceived competence in terms of offensive skills. These findings are consistent with those of antecedent research suggesting that defensemen tend to be larger and possess greater upper-body strength than forwards, which reinforces the former’s perception of strength and power. However, the tactical dimension of ice hockey competence produced an unexpected outcome insofar as it revealed that defensemen feel more competent than forwards when it comes to tactical abilities. A closer examination of the items forming the tactical dimension shows they include ease in the defensive zone, precise passes to teammates and the marking of opposing players. While these items can also be applied to forwards, they tend to be more directly related to defensemen. Thus, the role of defensemen is to prevent the opposing team from scoring and to initiate the attack by passing the puck to their forwards. In line with this hypothesis, the forwards’ primary objective is to score goals (or create situations that will lead to this). This explains their positive response to offensive abilities items such as puck handling, shooting accuracy and creativity in the offensive zone.

The fourth objective was to determine if there were differences in perceived competence among players based on their birth quartile. Our results show that early-born players tend to feel stronger, perceive themselves to have better tactical abilities and feel more coachable than those born later, partially confirming hypothesis 4. These results align with those of Lemoyne et al. (2021), who measured physical self-concept in a comparable sample. Young players born earlier in the selection year tend to have a physical advantage and therefore feel more competent than those born later. Players born earlier in the year also report higher levels of tactical ability and coachability. This may be partly because players born early in the selection year generally play more games and have more ice time, which offers them more opportunities to shine (Lemoyne et al., 2023). Moreover, coaches may trust these players more and feel they can implement game plans, thus positively affecting their perceived competencies.

### 4.1 Limitations and future directions

Although this study makes significant progress in evaluating how perceived competence is impacted by different variables of influence in ice hockey, some limitations prevail. First, the sample size consisted solely of adolescent male players, meaning results cannot be generalized to female ice hockey players. Since previous research also highlights potential gender differences in the way perceived competence is fostered during adolescence (Cairney et al., 2012), the inclusion of female hockey players in future studies may be an aspect to consider. Another limitation concerns the players’ position. In this regard, goaltenders were not included in the study as there is no reliable questionnaire that addresses their perception of competence. Since goaltenders play a crucial and equally important role in the hockey ecosystem, however, it would be interesting to examine their perceptions of their hockey competencies in future studies. Furthermore, information regarding indicators of early sport specialization was collected retrospectively, which increases the risk of recall bias (Raphael, 1987). A longitudinal study could be conducted to track groups of specialized and non-specialized athletes and measure the evolution of their perceived competence during their development and, potentially, into early adulthood when other consequences of specialization may arise. Possible selection bias must also be mentioned. Indeed, most of the best performing players in the U18 age category may play in the U18AAA league or the Canadian Hockey League and were not included in the recruitment process. Because the aforementioned leagues reunite Canada’s best prospects, these athletes may have reported higher perceived competence scores. Such a bias might eliminate some of the gap we observed between younger and older players. At the other of the spectrum, the present study covers players who were active at the time of data gathering. Thus, players who had given up ice hockey and had a potentially lower perceived competence were also excluded. Finally, the results presented here are specific to athletes in the Quebec development model, which hinders the generalizability of the results. In this regard, cross validation studies with other populations (age group, nationality, gender), could confirm whether the concept of perceived competence in ice hockey can be conceptualized similarly elsewhere.

### 4.2 Practical applications

The decline in perceived competence during adolescence could be mitigated by better coaching and support from coaches and other sports stakeholders. Toward the end of adolescence, athletes should, crucially, be made aware of their strengths and given opportunities to experience sports allowing them to leverage these strengths (Curran et al., 2016). This nurtures the sense of competence and provides a chance to develop in another sport should they decide to leave ice hockey. Results of the present study suggest that ESS measures are positively associated with perceived ice hockey competence. At first glance, it appears that players who are highly involved in their sport of choice develop a strong sense of perceived competence in it. Their choice may be an ill-advised one, however, as it could entail the risk of overuse injuries (Bell et al., 2018) and other potential psychosocial consequences (Gould, 2010). A sport specialization environment that enhances players’ positive development is therefore important (Holt et al., 2020). Thus, an option worth considering is participation in summer development camps ideally supervised by professionals who create a healthy motivational climate (Allen and Hodge, 2006) and allow players to develop their gross motor skills (Jaakkola et al., 2017). Results regarding the third objective suggest that later-born players may be disadvantaged by the impacts this has on perceived hockey competence. To counter these negative impacts, coaches – especially those responsible for most competitive teams/programs – should be advised to take the situation into account during team selection. Additionally, the governing bodies of sports federations should also consider the idea of incorporating teams composed of players born in the latter part of the year in tournaments or exhibitions held at the end of the season. As well, they should acknowledge the performances of later-born athletes in public publications. Regarding the fourth objective, perceived competence appears to differ based on the players’ position. Since the positions of forwards and defensemen correspond to different components of perceived competence, young players may do well to vary their position during the season and develop abilities that fit the requirements of each position. Indeed, Hockey Canada recommends this course of action for players under the age of twelve, as it allows them to expand their repertoire of skills and improve their knowledge and understanding of their sport (Hockey Canada, 2023). Furthermore, coaches should emphasize the importance of tactical skills and physical involvement in forwards, while encouraging defenders to contribute more positively to the attack to improve players’ perception of competence.

## 5 Conclusion

This study explores the impact of relative age, early specialization and position selection on perceived competence in Canadian minor ice hockey. This research highlights the fact that players in their late teens tend to have lower perceived competence in ice hockey than those in their early teens. Next, early sport specialization behaviors in ice hockey are positively associated with perceived competence in ice hockey as well as PSC. The study also shows that defensemen perceive themselves as more competent in tactical and physical aspects, while forwards feel more competent in their offensive abilities, demonstrating differences in relation to player position. Finally, a relative age effect is present in the sample studied, as players born earlier in the selection period feel more competent in terms of power, tactical abilities and coachability. The findings shed light on the complex interplay between individual factors and perceived competence in the context of Canadian minor ice hockey.

## Data availability statement

The raw data supporting the conclusions of this article will be made available by the authors, without undue reservation.

## Ethics statement

This study involving human/animal participants was reviewed and approved by the ethics committee of the Université du Québec à Trois-Rivières. The studies were conducted in accordance with the local legislation and institutional requirements. Written informed consent for participation in this study was provided by the participants' legal guardians/next of kin.

## Author contributions

VH: Data curation, Funding acquisition, Investigation, Methodology, Visualization, Writing – original draft. JL: Formal analysis, Supervision, Writing – review & editing.
