# Supplementary material for: Perceived competence in ice hockey and its associations with relative age, early sport specialization, and players’ position
Source: Front Psychol. 2024 Jan 25;15:1336529. doi: 10.3389/fpsyg.2024.1336529 (PMC10850288; doi:10.3389/fpsyg.2024.1336529)
Supplement: Supplementary file 1 [file Table_1.DOCX]

**Appendix A.** Self-Perceived Ice Hockey Competence Scale items

| Original items | English translation | | |
| --- | --- | --- | --- |
|  |  |  |  |
| 1. Je lance avec puissance. | *I have a powerful shot* | | |
| 2. Mes tirs au but sont précis. | *I have an accurate shot* | | |
| 3. J’excelle dans le maniement de la rondelle. | *I am good at stickhandling* | | |
| 4. J’effectue des passes précises à mes coéquipiers. | *I make accurate passes to my teammates* | | |
| 5. Je patine rapidement. | *I am a fast skater* | | |
| 6. Je me déplace avec agilité sur la patinoire. | *I move around the rink with agility* | | |
| 7. Je m’implique physiquement sur la glace. | *I am physically involved on the ice* | | |
| 8. Je suis endurant au niveau cardiovasculaire. | *I have good endurance/stamina* | | |
| 9. Je suis fort physiquement. | *I am physically strong* | | |
| 10. J’ai des habitudes de travail qui m’aide à performer. | *I have a good work ethic that helps me to perform* | | |
| 11. Je vois les critiques de mon entraineur comme une occasion de m’améliorer. | *I see my coach’s comments as an opportunity to improve* | | |
| 12. J’influence positivement mes coéquipiers. | *I am a positive leader to my teammates* | | |
| 13. J’accepte le rôle que l’entraineur me donne au sein de l’équipe. | *I accept the role the coach gives me within the team* | | |
| 14. Je gagne mes batailles à 1 contre 1. | *I win my one-on-one* *battles* | | |
| 15. Je prends des décisions intelligentes sans la rondelle. | *I take good decision without the puck* | | |
| 16. Je suis créatif sur la glace. | *I am creative on the ice* | | |
| 17. Je peux appliquer les systèmes de jeux sans problème. | *I can apply the game systems without problem* | | |
| 18. Je suis efficace en zone défensive. | *I am efficient in the defensive zone* | | |
| 19. Je suis efficace en zone offensive. | *I am efficient in the offensive zone* | | |
| 20. Je reste confiant même si mon temps de jeu diminue. | *I stay confident even if playing time is diminished* | | |
| 21. Je suis bon dans les replis défensifs. | *I* *am good at back checking* | | |
| 22. Quand j’ai des difficultés je ne me décourage pas ou pas longtemps. | *I am not easily discouraged, even in difficult situation* | | |
